# Primary Pulmonary Hodgkin Lymphoma Simulating a Mediastinal Tumour: an Uncommon Occurrence

**DOI:** 10.4084/MJHID.2013.013

**Published:** 2013-02-16

**Authors:** Stefano Fratoni, Elisabetta Abruzzese, Pasquale Niscola, Malgorzata Monika Trawinska, Edoardo Mercadante, Andrea Casullo, Paolo de Fabritiis, Alessio Perrotti, Giuseppe Santeusanio

**Affiliations:** 1 Pathology Department, S. Eugenio Hospital, Rome (Italy); 2 Hematology Department, S. Eugenio Hospital, Rome (Italy); 3 Department of Thoracic Surgery, S. Eugenio Hospital, Rome (Italy); 4 Department of Radiology, S. Eugenio Hospital, Rome (Italy)

## Abstract

The case of a patient with primary pulmonary Hodgkin Lymphoma simulating a mediastinal tumour is reported for its rarity and the diagnostic concerns encountered by us.

## Introduction

Although the secondary infiltration of lung parenchyma by Hodgkin’s lymphoma (HL) may occur, pulmonary HL occurred as an isolated tumour (primary pulmonary HL, PPHL) represents an uncommon observation.^1^–^6^ A case of PPHL simulating a mediastinal tumour is herein reported with a brief discussion on the most important concerns encountered during the diagnostic process.

## Case Report

**Figure 1:**
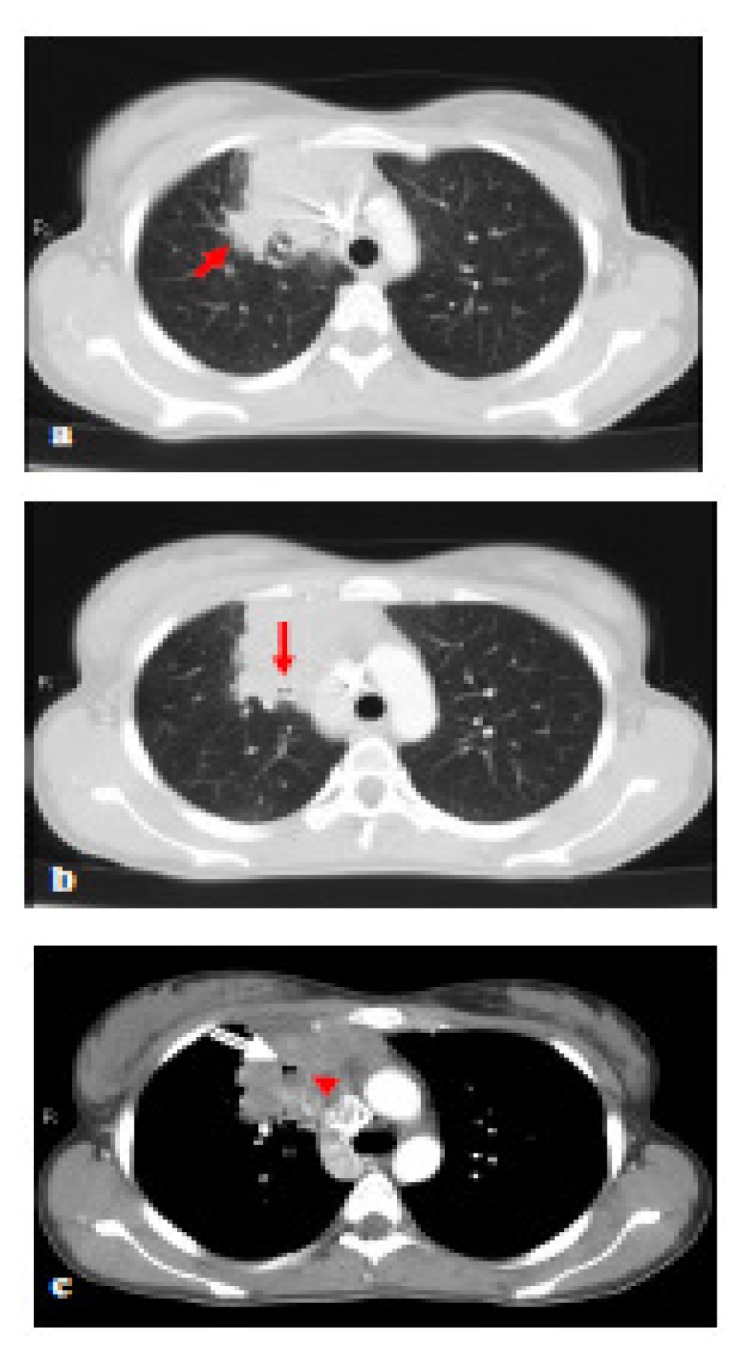
Chest CT scan at onset. A. Parenchymal lung involvement by pathologic tissue (Hodgkin’s lymphoma). The pulmonary origin is demonstrated by the irregular margins of the lesion, especially evident in correspondence with the posterolateral portion (arrow). B. Intralesional bronchial structures (arrow). C. Window to the mediastinum. It is evident the "angiogram sign" (arrowhead) in the context of the parenchymal mass. Also here is evident an intralesional bronchial branch (arrow).

**Figure 2:**
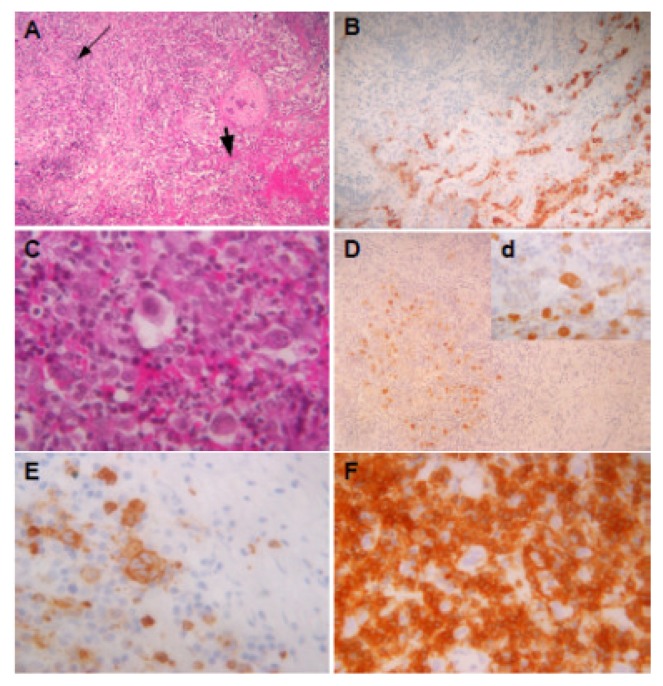
Microscopic examination at diagnosis. Microscopic examination shows pulmonary tissue (A - indicated by a short arrow) with intra-parenchimal nodule of Hodgkin’s Lymphoma (A - indicated by a thin arrow). At high magnification the nodule shows (C) many typical lacunar variant of Hodgkin’s cells. Immunohistochemical analysis shows the expression of Surfactant Apoprotein-A in lung tissue (B) and staining of CD30 (D and d) and CD15 (E) antibodies in Hodgkin’s cell surrounded by rosettes of CD3+ T lymphocytes (F).

The patient was a 27-year-old non-smoker woman who kept under our attention because a mediastinal enlargement incidentally discovered by a standard thoracic radiography performed because of a vague pulmonary symptomatology, consisting of dry cough and mild dyspnoea on exertion. A comprehensive diagnostic work-up was performed. Routine laboratory analysis showed no abnormal findings, with the exception of a slight neutrophilic leukocytosis. Moreover, all common virus infections, including HIV, were ruled out by specific antibody tests. A computed tomography (CT) scan of the chest revealed a pulmonary mass (8 cm in diameter) in the middle lobe of right lung; this mass reached out to anterior-medium mediastinum, simulating a mediastinal neoplasm. No findings consistent with any pulmonary infiltrates or hilar and mediastinal lymph nodes involvement were detected (Figure 1). On the mediastinoscopy, a lymphoproliferative neoplasm was highly suspected; however, the pathological specimens taken during an excision biopsy performed during an anterior mediastinotomy was unsuitable for a histological analysis. Therefore, a video-assisted thoracoscopy (VATS) with intraoperative biopsy were performed. VATS showed a solitary nodular mass confined to the lung and revealed the absence of any mediastinal invasion, as well as no extension into the pleura and the anterior chest wall. The microscopic examination (Figure 2) revealed lung tissue effaced by a nodular growth with thick collagen bands. Such nodules showed a dense cellular infiltrate composed of polymorphous inflammatory cells. At high magnification, many clusters of “lacunar cells”, with a characteristic cytoplasm retraction artefact and enlarged mono or multilobated nuclei with pale chromatin, surrounded by CD3+ T lymphocytes, were observed. Immunohistochemical analysis revealed para-nuclear and membrane expression of CD30 and CD15 antigens, weak nuclear expression of PAX5 (BSAP) while Leukocyte Common Antigen (LCA) CD45RB, CD20, CD3, Epithelial Membrane Antigen, ALK(p80) and Epstein-Barr virus (LMP1) antibodies were not expressed. These findings were consistent with a diagnosis of classic HL, nodular-sclerosis subtype grade 2 (Figure 2).

A comprehensive staging work up, including bone marrow trephine biopsy and a body CT scan, excluded any other extra-pulmonary disease localizations. No B symptoms were complained by the patient. So that a diagnosis of PPHL, stage IAE (L) according to the Ann-Arbor Staging System was made. The patient was treated with 6 courses of ABVD (doxorubicin, bleomycin, vinblastine and dacarbazine) regimen, achieving the complete disappearance of the pulmonary mass. Follow up at 1, 2 and 5 years showed a negative CT and Positron Emission Tomography scans, confirming the complete remission of the disease. To date, the patient is well; in the meantime, she had delivered two healthy baby boys.

## Discussion

The case here reported is that of an unusual form of a PPHL which didn’t show the most common radiologic features described in other reported observations. Indeed, PPHL is usually characterized by multiple nodules, reticolonodular shadows or mass lesion;^2^ unlike what is reported in literature,^1^–^6^ our patient presented a pulmonary lesion of the right middle pulmonary lobe growing into the mediastinum, simulating an exclusively mediastinal tumour, in the absence of hilar node involvement. Our patient has been cured by a standard chemotherapy, reflecting our findings the relatively good prognosis of PPHL.^1^ Therefore, the early recognition of PPHL and the differential diagnosis between it and other tumours of the lung and mediastinum have a critical value. With this regard, our experience outlined the diagnostic role of VATS,^7^ suggesting our experience that the use of frozen section and the thoracoscopic approach should guide the surgeon to obtain a representative biopsy of the lesion in order to perform both an optimal paraffin histology and immunohistochemical studies, allowing an appropriate differential diagnosis. With this regard, differential diagnosis between PPHL and other thoracic diseases associated with mediastinal enlargements^7^–^10^ may troublesome and includes infectious and non-infectious granulomatous diseases, reparative phenomena and solid tumours. Granulomatous inflammation, when associated with HL, can be similar to other granulomatous lesions, such as Wegener granulomatosis and mycobacterial and fungal infections. The presence of the caseous necrosis, the identification of infecting organisms with appropriate special stain, the lack of Hodgkin’s cells and of vasculitic features associated with Wegener granulomatosis, can allow an appropriate differential diagnosis between these lesions. In addition, reparative phenomena, such as organizing pneumonia, can be similar to HL; however, in these occurrences, classic Reed-Sternberg or atypical mononuclear Hodgkin cells are obviously absent. Again, other lymphoproliferative disorders such as mediastinal HL and others non-Hodgkin lymphomas should be excluded. In particular, the nodular sclerosis subtype of classic HL often arises in tracheobronchial or mediastinal lymph nodes and subsequently can infiltrate the lung. The most useful criteria that allows to distinguish PPHL from nodal HL involving the lung is the exclusion of extrapulmonary HL, since PPHL is confined to the pulmunary site with a propensity to arise in the middle or upper portion of the lung. Lastly, differential diagnosis with non-Hodgkin lymphomas or other malignancy is easily prompted by a careful evaluation of morphologic, immunoistochemical features and clinical data.

Therefore, our experience suggested that an integrated clinicopathologic and surgical approach can be necessary to make a diagnosis of PPHL, which should be included into the differential diagnosis of any suspicious mediastinal enlargement.
